# Fraxinellone Induces Hepatotoxicity in Zebrafish through Oxidative Stress and the Transporters Pathway

**DOI:** 10.3390/molecules27092647

**Published:** 2022-04-20

**Authors:** Shuting Wang, Jie Bao, Jie Li, Wanfang Li, Mengyin Tian, Caixia Qiu, Fei Pang, Xin Li, Jianbo Yang, Yuchi Hu, Sujuan Wang, Hongtao Jin

**Affiliations:** 1 Institute of Materia Medica, Chinese Academy of Medical Sciences & Peking Union Medical College, Beijing 100050, China; wangshuting@imm.ac.cn (S.W.); baojie@imm.ac.cn (J.B.); lijie@imm.ac.cn (J.L.); liwanfang@imm.ac.cn (W.L.); tammy199501@126.com (M.T.); qiucaixia@imm.ac.cn (C.Q.); pangfei@imm.ac.cn (F.P.); lixin@imm.ac.cn (X.L.); 2 Beijing Union-Genius Pharmaceutical Technology Co., Ltd., Beijing 100176, China; 3 Institute for Control of Chinese Traditional Medicine and Ethnic Medicine, National Institutes for Food and Drug Control, Beijing 100050, China; yangjianbo@nifdc.org.cn; 4 NMPA Key Laboratory for Safety Research and Evaluation of Innovative Drug, Beijing 100050, China; yuchihu@bidc.org.cn

**Keywords:** fraxinellone, zebrafish, hepatotoxicity, transporter, metabolomics

## Abstract

Fraxinellone (FRA), a major active component from Cortex Dictamni, produces hepatotoxicity via the metabolization of furan rings by CYP450. However, the mechanism underlying the hepatotoxicity of FRA remains unclear. Therefore, zebrafish larvae at 72 h post fertilization were used to evaluate the metabolic hepatotoxicity of FRA and to explore the underlying molecular mechanisms. The results showed that FRA (10–30 μM) induced liver injury and obvious alterations in the metabolomics of zebrafish larvae. FRA induces apoptosis by increasing the level of ROS and activating the JNK/P53 pathway. In addition, FRA can induce cholestasis by down-regulating bile acid transporters P-gp, Bsep, and Ntcp. The addition of the CYP3A inhibitor ketoconazole (1 μM) significantly reduced the hepatotoxicity of FRA (30 μM), which indicated that FRA induced hepatotoxicity through CYP3A metabolism. Targeted metabolomics analysis indicates the changes in amino acid levels can be combined with molecular biology to clarify the mechanism of hepatotoxicity induced by FRA, and amino acid metabolism monitoring may provide a new method for the prevention and treatment of DILI from FRA.

## 1. Introduction

Drug-induced liver injury (DILI) is one of the major concerns for the safe use of drugs, recently for herbal medicines in particular. The annual incidence of DILI continues to increase in countries with high usage of herbal medicines, including China, Japan, and the United States [1,2,3]. The toxic chemical ingredients of herbs can cause idiosyncratic, intrinsic, or mixed liver injury, inducing oxidative stress either directly or indirectly (metabolic activation) and the activation of various signal transduction pathways, resulting in mitochondrial dysfunction and an imbalance in bile acid homeostasis. In addition, a large part of preclinical evaluation indices fail to accurately describe and reveal the clinical features of herbal medicines, thereby increasing the challenge of herbal and traditional medicine development and clinical safe use [2,4]. Cortex Dictamni (CD) is the root cortex of *Dictamnus dasycarpus* Turcz, a perennial herb in the Rutaceae family used in traditional medicine for the treatment of inflammation, bacterial infection, and allergies [5,6]. However, CD is toxic to the liver, with clinical manifestations similar to those of acute liver cell damage, abnormal liver function, jaundice, cholestasis, and chronic hepatitis with fibrosis, as well as other pathological changes [7,8,9]. It has been reported that bioactivation mediated by metabolic enzymes is the main mechanism underlying CD-induced DILI [10,11]. Several furan ring compounds represented by fraxinellone (FRA) are the main active components extracted from CD, and their furan ring functional groups can be metabolized by cytochrome P450 (CYP450) to form a *cis*-butene structure, which leads to hepatotoxicity [11]. FRA can significantly increase the levels of serum ALT and AST in mice, and the addition of ketoconazole (KCZ) can reduce the hepatotoxicity of FRA, suggesting that FRA has a warning effect on the structure of the furan ring [12]. However, the current research on the mechanism of hepatotoxicity of FRA is limited to the detection of pharmacokinetics in vivo and the structural identification of the main metabolic groups in the liver microsome system [12]. Therefore, zebrafish, a convenient model organism, is selected in this study. The aim is to find the possible toxic pathways and metabolic markers in vivo based on the study of the molecular mechanism of metabolic activation, so as to lay the foundation for safe clinical use.

The zebrafish (*Danio rerio*) is a widely used model organism to test the toxicity of various compounds [13]. As an efficient and convenient animal model, zebrafish have the advantages of a short reproductive period, high fecundity, body transparency, and the ability to observe tissues and organs by light microscopy [14,15]. Additionally, the zebrafish genomes are orthologs with the human CYP450 genes, especially for the CYP1–3 families, which are primarily responsible for drug metabolism, with similar induction mechanisms in nuclear hormone receptors and CYP3A subfamilies as humans. Notably, the mechanisms underlying liver development and bile formation in zebrafish are similar to those in mammals, as characterized by enriched transporter expression in the liver and intestine [16,17]. These transporters distributed in the apical and basolateral membranes of zebrafish hepatocytes regulate the efflux of bile into cholangiocytes and the reabsorption of blood, thereby playing an important role in maintaining bile acid homeostasis [18,19,20]. At present, some immortalized cell lines, such as HepG2 liver cancer cells, are slightly less sensitive in evaluating CYP inductions and often need to use derived cells expressing cytochrome P450s or combined with human liver microsomes to predict DILI, leading to high cost and low accuracy [21,22,23,24]. Based on the above knowledge, zebrafish may be a suitable and comparatively inexpensive animal model for the study of metabotropic DILI.

Targeted amino acid metabolomics is a method that quantitatively measures specified metabolites in biological systems (cell models, tissues, organs, or whole organisms) and the dynamic changes of these metabolites under various stimuli [25,26]. It is an omics technology developed after genomics, transcriptomics, and proteomics. The liver is an important organ for amino acid metabolism and plays an important role in the balance of amino acid metabolism [27,28,29]. When hepatocytes are seriously injured, it will lead to amino acid metabolism disorders, especially a change in the ratio of branched chain amino acids (BCAAs) to aromatic amino acids [30]. Therefore, the amino acid level may be a good biomarker of DILI, and the study of amino acid metabolic networks may be helpful to evaluate liver function. Metabolomics is used to understand whether DILI has special metabolite characteristics which can be distinguished from healthy controls. In addition, the differences in metabolic characteristics and the amino acids of metabolites among groups can also explain the different mechanisms of herbal-medicine-induced DILI [26,31].

Based on the known metabolic toxicity of FRA and the application advantages of zebrafish in drug safety evaluations, the study used 72 h post fertilization (hpf) zebrafish larvae to evaluate the hepatotoxicity of FRA by the determination of the survival rate, microscopic evaluation of the hepatotoxicity, including liver gray value assay, AO staining, liver biochemical assay, histopathological analysis, together with ROS assay by flow cytometry. qRT-PCR analysis and western blot analysis of the targeted amino acid metabolomics were carried out to evaluate the mechanic pathways. The inhibitory effects of KCZ on FRA-induced hepatotoxicity were performed to validate the results.

## 2. Results

### 2.1. Effects of FRA on Survival of Zebrafish Larvae

**Figure 1:**
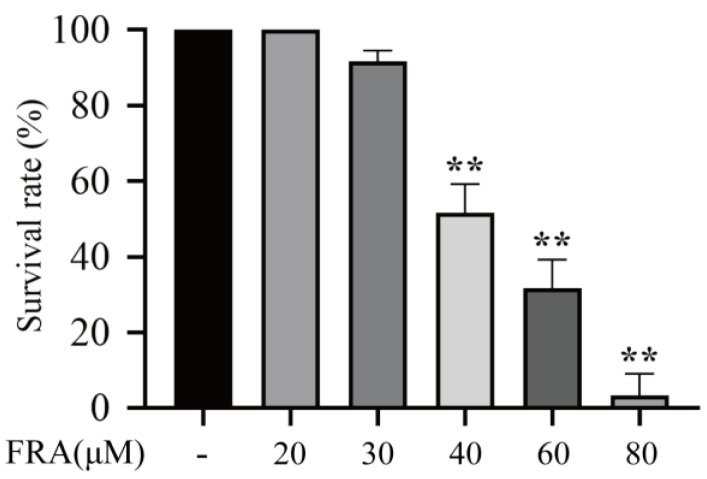
Survival rate of zebrafish larvae exposed to FRA for 48 h. Data are presented as the mean ± SD. Each experiment was performed 3 times with 30 randomly selected zebrafish larvae. ** *p* < 0.01 vs. control.

The zebrafish larvae were treated with 20, 30, 40, 60, or 80 μΜ FRA for 48 h, and the number of deaths was observed with an aquatic biomicroscope. The results showed that the LD_50_ and LD_10_ values were about 46.91 and 32.39 μM, respectively (Figure 1).

### 2.2. Hepatotoxic Effects of FRA on Zebrafish Larvae

**Figure 2:**
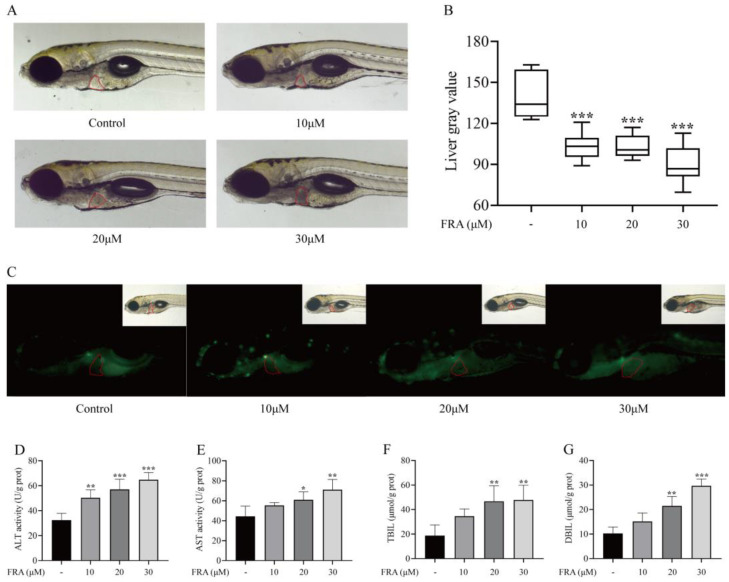
Hepatotoxic effects of FRA on zebrafish larvae. Treatment with different concentrations of FRA for 48 h blackened the liver area of zebrafish larvae (**A**) and increased the gray value (**B**). AO staining showed that the proportion of apoptotic hepatocytes had increased (**C**). Changes to levels of ALT (**D**), AST (**E**), TBIL (**F**), and DBIL (**G**) in zebrafish larvae treated with FRA for 48 h. Data are presented as the mean ± SD. Each experiment was performed 3 times with 40 randomly selected zebrafish larvae. * *p* < 0.05 vs. control; ** *p* < 0.01 vs. control; *** *p* < 0.001 vs. control.

With the sublethal dose of 30 μΜ (LD_10_) as the upper limit, a series of FRA concentrations was used to evaluate the hepatotoxicity to zebrafish under an ultra-depth-of-field microscope. The liver of normal zebrafish larvae is relatively transparent under the microscope, but after treatment with hepatotoxic drugs, the texture of the liver tissue is amorphous, the structure is disordered, and the color turns black, indicating liver degeneration. After treatment with 10, 20, or 30 μΜ FRA for 48 h, the zebrafish liver area became black, and the gray value decreased with the increase in FRA concentration (Figure 2A,B). In apoptotic cells, apoptotic bodies are formed due to chromatin pyknosis or their breakage into fragments of different sizes, which will be stained by AO into dense yellowish green fluorescence. Under a fluorescence microscope, AO staining showed that the fluorescence intensity of zebrafish larvae exposed to FRA increased, and the proportion of apoptotic cells was higher than that of the blank control group (Figure 2C). Additionally, the levels of ALT, AST, TBIL, and DBIL in the supernatant of zebrafish homogenate were significantly increased in a concentration-dependent manner (Figure 2D–G). Those results indicate that FRA have strong hepatotoxicity to zebrafish larvae.

### 2.3. Histopathological Evaluation of FRA-Induced Hepatotoxicity

**Figure 3:**
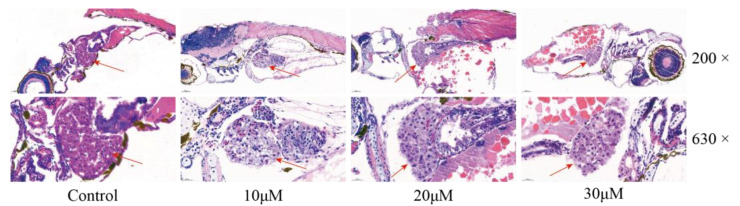
Effects of FRA on liver morphology. The liver is indicated with red arrows. Low-magnification images were captured at 200× and high-magnification images at 630×.

Histopathological evaluation of FRA-induced hepatotoxicity was conducted by staining with hematoxylin and eosin (H&E) (Figure 3). Imaging at low magnification confirmed that the liver was located in the zebrafish’s abdominal cavity. The surrounding intestine and pancreas were also visible. At high magnification, zebrafish hepatocytes in the blank control group were intact in a tight and regular arrangement, and the nuclei were rounded and located in the centers of the cells. However, administration of FRA induced swelling of the zebrafish hepatocytes with crowding of the nuclei to one side of the cell due to steatosis, vacuolation of the cytoplasm of the hepatocytes, and punctate or focal necrosis, showing the typical manifestations of liver injury.

### 2.4. Effects of FRA on ROS Levels of Zebrafish Larvae

**Figure 4:**
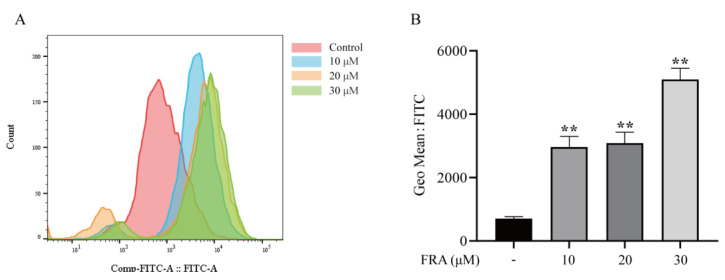
At 48 h after FRA treatment, flow cytometry was performed to determine ROS levels (**A**) and average fluorescence intensity (**B**). Data are presented as the mean ± SD. Each experiment was performed 3 times with 50 randomly selected zebrafish larvae. ** *p* < 0.01 vs. control.

ROS are formed in cells during normal metabolism. When cells are damaged by internal and external factors, the production of ROS increases, which destroys the balance between oxidation and the antioxidant system and finally leads to excessive oxidation. To confirm that FRA causes oxidative stress in hepatocytes, ROS levels were measured. The results showed that the ROS levels were significantly increased in the FRA treatment groups, and the ROS levels in the high dose group were increased nearly 3-fold compared with the control group (Figure 4A,B).

### 2.5. Effects of FRA on the Expression of Bile Acid Transporters and JNK/p53 Pathway

**Figure 5:**
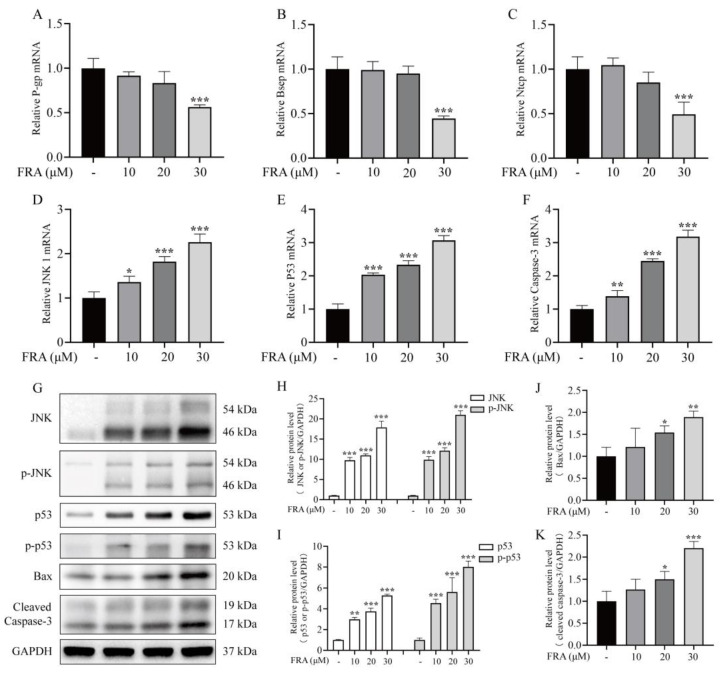
Effects of FRA on the expression levels of bile acid transporters and components of the JNK/p53 pathway. FRA inhibited mRNA expression of the bile acid transporters P-gp (**A**), Bsep (**B**), and NTCP (**C**), and increased those of the apoptotic genes JNK1 (**D**), caspase-3 (**E**), and p53 (**F**). The protein levels of JNK, p-JNK (**H**), p53, p-p53 (**I**), Bax (**J**), and cleaved caspase-3 (**K**) were determined by western blot analysis. GAPDH was used as an internal reference to normalize the results (**G**). Data are presented as the mean ± SD. Each experiment was performed 3 times with 40 randomly selected zebrafish larvae. * *p* < 0.05 vs. control; ** *p* < 0.01 vs. control; *** *p* < 0.001 vs. control.

To further explore the mechanism underlying liver injury induced by FRA, the expression levels of genes involved in maintaining bile acid homeostasis (P-gp, Bsep and Ntcp) and apoptosis regulation (JNK1, p53, and caspase-3) were measured by qRT-PCR. The results showed that compared with the control group, the mRNA expression levels of Bsep, P-gp and Ntcp in the high dose group of FRA decreased nearly 50% (Figure 5A–C), and the mRNA expression levels of JNK1, caspase-3, and p53 increased more than two-fold (Figure 5D–F). The protein expression levels of JNK, p53, and caspase-3 were determined by western blot analysis. The results showed that the levels of phosphorylated JNK and p53 increased significantly in a concentration-dependent manner. Moreover, the protein levels of Bax and cleaved caspase-3, as downstream markers of the JNK pro-apoptotic pathway, had increased with increased levels of phosphorylated JNK (Figure 5G–K). The JNK signal transduction pathway is an important branch of the MAPK pathway. Compared with the control group, the levels of phosphorylated JNK in the FRA group increased, which promoted the expression of the apoptotic protein Bax in mitochondria and further promoted cell apoptosis.

### 2.6. Inhibitory Effects of KCZ on FRA-Induced Hepatotoxicity

**Figure 6:**
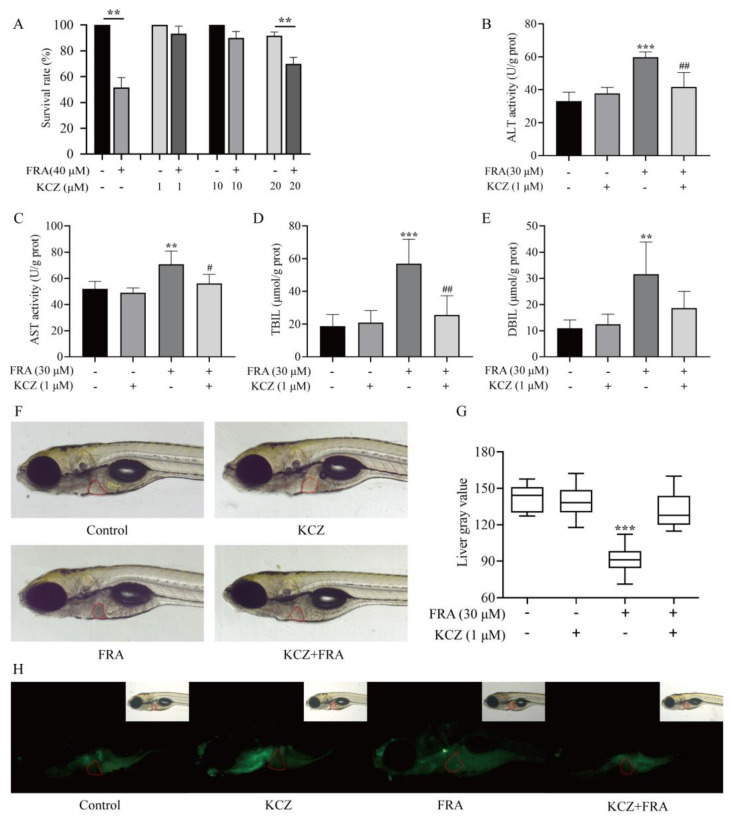
Inhibitory effects of KCZ against FRA-induced hepatotoxicity. (**A**) KCZ reduced FRA-induced mortality of zebrafish larvae. The biochemical indices of ALT (**B**), AST (**C**), TBIL (**D**), and DBIL (**E**) decreased significantly after KCZ treatment. (**F**–**H**) Morphological observation and AO staining to determine the degree of liver injury and apoptosis. Data are presented as the mean ± SD. Each experiment was performed 3 times with 40 randomly selected zebrafish larvae. ** *p* < 0.01 vs. control; *** *p* < 0.001 vs. control; ^#^ *p* < 0.05 vs. FRA + KCZ; ^##^ *p* < 0.01 vs. FRA+KCZ.

Under the same administration conditions, FRA-induced mortality was significantly decreased with the addition of different concentrations of KCZ (Figure 6A). Based on the experimental results, 1 μΜ KCZ was selected as a safe dose for follow-up mechanism verification. After coadministration for 48 h, ALT and AST levels in the KCZ co-treatment group significantly decreased compared with the FRA treatment group, the levels of TBIL and DBIL decreased nearly 50%, and there were no significant differences as compared with the blank control group (Figure 6B–E). Microscopic observation showed that the liver gray value of zebrafish larvae in the CYP3A inhibitor KCZ co-treatment group returned to normal, and AO fluorescence staining showed that the proportion of apoptotic cells decreased (Figure 6F–H).

### 2.7. KCZ Altered the Expression Patterns of Transporter and Apoptotic Proteins Inhibited by FRA

**Figure 7:**
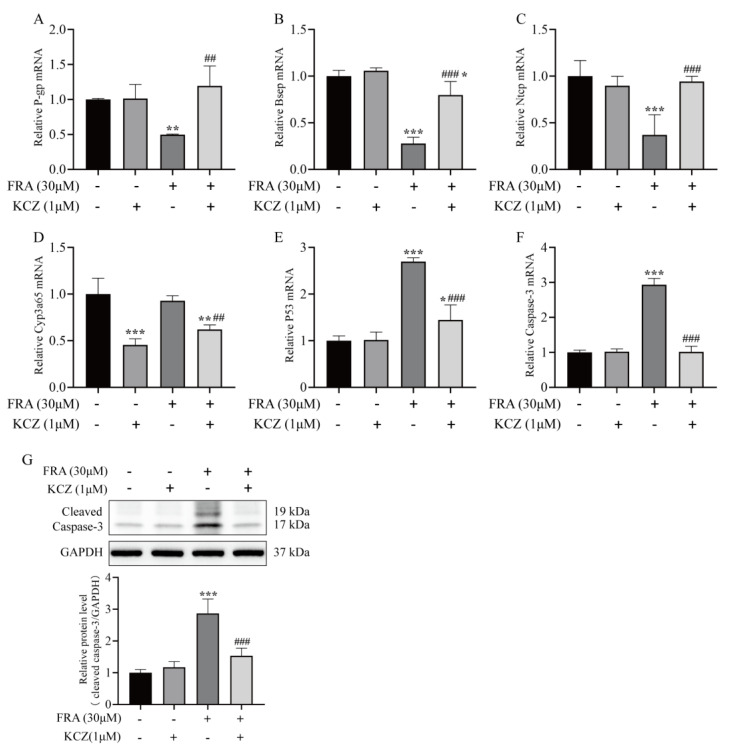
KCZ altered the expression patterns of transporter and apoptotic proteins inhibited by FRA. The mRNA expression levels of P-gp (**A**), Bsep (**B**), Ntcp (**C**), Cyp3a65 (**D**), p53 (**E**), and caspase-3 (**F**) were detected by qRT-PCR analysis. (**G**) The protein expression level of cleaved caspase-3 was detected by western blot analysis. Data are presented as the mean ± SD. Each experiment was performed 3 times with 40 randomly selected zebrafish larvae. * *p* < 0.05 vs. control; ** *p* < 0.01 vs. control; *** *p* < 0.001 vs. control; ^##^ *p* < 0.01 vs. FRA + KCZ; ^###^ *p* < 0.001 vs. FRA + KCZ.

KCZ altered the gene expression patterns of metabolizing enzymes and transporters associated with FRA-induced liver injury and cholestasis. The qRT-PCR and western blot results showed that KCZ restored the mRNA expression levels of *Bsep*, *P-gp*, and *Ntcp* inhibited by FRA and significantly decreased *cyp3a65* mRNA levels as compared with the FRA treatment group (Figure 7A–D). These findings suggest that KCZ can reduce the production of the toxic metabolites of FRA by inhibiting expression of members of the CYP3A subfamily. Additionally, compared with the FRA treatment group, the mRNA expression of *p53* and *caspase-3* in the KCZ co-treatment group significantly decreased, and cleaved caspase-3 protein expression decreased by 50%. In addition, there was no significant difference with the blank control group, which confirmed that the CYP3A inhibitor KCZ ameliorated the toxicity of FRA (Figure 7E–G).

### 2.8. KCZ Restored Amino Acid Metabolism Altered by FRA

**Figure 8:**
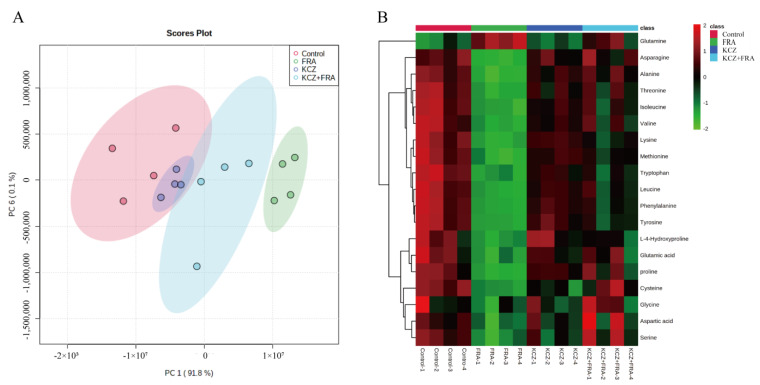
KCZ restored the amino acid metabolism spectrum altered by FRA. (**A**) PCA scores of zebrafish larvae samples in the control, FRA, KCZ, and KCZ and FRA groups. The 95% confidence intervals are shown. (**B**) A heatmap showing changes in the contents of 19 amino acids related to zebrafish liver injury obtained by GC-MS/MS. Each experiment was performed 3 times with 40 randomly selected zebrafish larvae.

To further verify the hepatotoxicity of FRA and the toxicity inhibition of KCZ, targeted metabolomics analysis was conducted to detect dynamic changes to amino acids in zebrafish. In the PCA score map, the comprehensive variables of the main metabolites in the FRA treatment group, KCZ treatment group, and FRA and KCZ co-treatment group were highlighted in green, blue, and purple, respectively, compared with the control group in the red area. The results showed that, within the 95% confidence interval, the KCZ treatment group and the blank control group had the most overlap, while the FRA treatment group and the blank control group were clearly separated by significant differences. As compared with the FRA treatment group, the amino acid metabolites of the KCZ and FRA co-treatment group showed a tendency for callback and partially overlapped with the blank control group (Figure 8A). A heatmap showed that the contents of cysteine, glutamic acid, leucine, and tryptophan in zebrafish significantly decreased after FRA treatment, while the detoxification product glutamine significantly increased. After KCZ co-treatment, the expression levels of these amino acids were largely restored (Figure 8B).

## 3. Discussion

**Figure 9:**
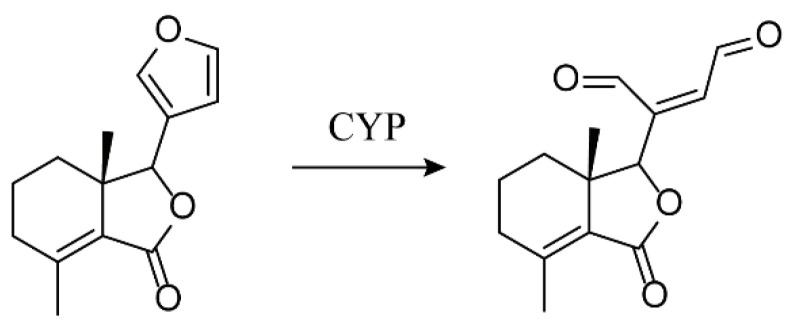
Chemical structures of fraxinellone (FRA) and its metabolites.

Some furans produce hepatotoxicity through activation of metabolizing enzymes, but structural differences will lead to different toxicity mechanisms [32,33,34]. Considering the furan ring functional group of FRA, zebrafish larvae were used as a sensitive in vivo model to evaluate the hepatotoxicity of FRA (Figure 9). The mechanism underlying the toxicity of FRA was also investigated by a series of molecular biology techniques and metabolomics targeting changes in amino acid profiles.

The secretion of bile is the result of the vector transfer of osmotic active substances from hepatocytes to bile ducts, mostly occurring on the tubular membrane of hepatocytes [35]. Some transporters are involved in these transmembrane processes, such as bile salt, bilirubin, cholesterol, and lipid transporters [36]. The dysfunction of these transporters will lead to the disturbance of the bile flow of hepatocytes and intrahepatic bile duct, resulting in the increase in the bilirubin and bile salt levels in the liver and blood, as well as the imbalance of the lipid metabolism, thus promoting cholestatic hepatocyte injury [37,38]. Bsep, P-gp, and Ntcp are the main protein transporters of bile acid and maintain bile acid homeostasis [39]. P-gp and Bsep are the key steps for bile acid to flow out of the tubule membrane and drive the enterohepatic circulation of bile acid, while Ntcp mediates the uptake of other substrates such as bile salts, sulfate compounds, drugs, and toxins via the basolateral membrane in a sodium-dependent manner. They maintain the balance of bile acids in the body by pumping bile acid from the blood back to the hepatocytes and transporting them from the hepatocytes to the cholangiocytes for efflux [35,40,41]. The results of the present study showed that FRA significantly reduced the mRNA levels of *Bsep*, *P-gp*, and *Ntcp* and increased the levels of ALT, AST, TBIL, and DBIL in zebrafish, which confirmed that FRA impedes the reabsorption of bile acid in the blood and the secretion of bile acid to the bile duct. Dysregulation of the function or expression of these transporters leads to the accumulation of bile acid in cells, thus promoting cholestatic hepatocyte injury.

In this study, the effect of FRA on the production of ROS in zebrafish was evaluated. ROS is a key initiating factor of oxidative stress. Excessive production of ROS in the body can induce oxidative stress, which will adversely affect macromolecules such as lipids, proteins, and nucleic acids. These macromolecules can directly cause inflammation and apoptosis and lead to toxic reactions [42,43]. ROS also supports the activation of the JNK pathway [44]. Phospho-JNK is transferred to the nucleus as a dimer to induce phosphorylation of the transcription factor p53, resulting in the accumulation and activation of p53 in response to DNA damage and the subsequent cell cycle arrest or apoptosis. Members of the Bcl-2 family regulate apoptosis induced by the intrinsic apoptotic pathway by controlling the permeability of the mitochondrial membrane. When DNA is damaged, activated p53 induces transcription of the pro-apoptotic protein Bax in the Bcl-2 family and forms oligomers that are translocated from the cytoplasm to the mitochondrial membrane, which increases membrane permeability, resulting in the release of cytochrome C from the mitochondria and the subsequent activation of the caspase pathway to initiate apoptosis. The increased production of ROS caused by changes to the mitochondrial membrane potential and inflammatory stress continue to activate the JNK/p53 pathway through positive feedback, ultimately resulting in irreversible apoptosis [45,46]. In this study, FRA caused an increase in the adaptability of JNK and mitochondrial apoptosis-related proteins and genes, resulting in excessive accumulation of ROS. The JNK/p53 signaling pathway activated by ROS may be involved in FRA-induced liver injury and oxidative stress in zebrafish.

It has been reported that some monomers extracted from CD have a furan ring structure, which is a typical metabolizable functional group, and it is speculated that the CD extract can be metabolized by CYP450 to produce hepatotoxic substances [11,12]. KCZ is a typical CYP3A inhibitor that is often used as an indicator of CYP3A inhibition in drug–drug interaction studies [47]. In the present study, KCZ significantly decreased the toxicity of FRA and induced changes to the gene expression of liver drug enzymes and transporters related to FRA-induced liver injury and cholestasis. This is consistent with the previous research results, where CYP3A4-mediated biological activation plays an indispensable role in in furan ring compounds induced hepatotoxicity [8,12]. The cytotoxicity was also positively correlated with the expression level of CYP3A4 [7]. In fact, many human CYP450 enzymes have direct homologous genes in zebrafish, which indicates that the metabolic spectrum of zebrafish may be similar to that of mammals [48,49]. The results showed that the zebrafish is not only a sensitive model to detect the hepatotoxicity of FRA in vivo, but also an appropriate model to explore the toxic activation of FRA mediated by CYP3A, which was rarely reported before. Compared with immortal cell lines with weak expression of CYP enzymes such as HepG2, zebrafish have a certain advantage in the screening of metabolic hepatotoxic drugs.

The liver is the main organ responsible for metabolism and plays an important role in amino acid homeostasis. Severe injury to hepatocytes causes amino acid metabolic disorders. The levels of branched chain amino acids (BCAAs) and aromatic amino acids are closely related to liver and metabolic diseases, especially. Glutathione, a tripeptide composed of glutamate, cysteine, and glycine, is the most abundant antioxidant in living organisms and mainly maintains cellular redox homeostasis [50,51]. Glutamate is an important substrate for the synthesis of glutathione and can be converted from glutamine, which has some effect on maintenance of the glutathione content. In this study, FRA significantly upregulated the content of glutamine in the tissues of zebrafish larvae, but its metabolite glutamate was downregulated, as was the content of cysteine, which explains the damage to the zebrafish liver caused by oxidative stress in response to increased ROS production. Studies have shown that glycine mediates inactivation of the JNK signaling pathway to reverse oxidative stress and inflammatory responses. Glycine can also significantly reduce expression levels of pro-apoptotic markers and increase protein expression of the anti-apoptotic protein Bcl-2 [52]. As compared with the control group, FRA significantly decreased the content of glycine in zebrafish, which was consistent with the upregulated activation of the JNK pathway and the apoptotic signaling found in this study.

Isoleucine, leucine, and valine are referred to as BCAAs due the presence of side chains with similar structures. Previous studies have shown that the lack of BCAAs in patients with liver cirrhosis is mainly due to the depletion of glutamate synthesis in skeletal muscles. BCAAs can promote the detoxification of ammonia to glutamine by correcting amino acid imbalances in the treatment of liver cirrhosis [53]. BCAAs can also reduce hepatic steatosis and liver injury by inhibiting Fas at the mRNA and protein levels [54]. In this study, as expected, along with liver injury induced by FRA, the contents of isoleucine, leucine, and valine were significantly downregulated, suggesting that BCAAs are potential markers of liver injury. FRA can cause other amino acid metabolic disorders, such as decreases in the contents of proline, alanine, tyrosine, tryptophan, and phenylalanine. Some studies have shown that alanine promoted recovery of liver damage and significantly reduced the levels of transaminase and TBIL in hepatotoxic rats. Tryptophan is also an essential amino acid that plays an important role in protein biosynthesis. Since most tryptophan is metabolized in the liver, the hepatotoxicity of most drugs will result in disorders of the tryptophan metabolism. Proline can restore serum levels of liver injury markers, reduce pathological damage to liver tissue, and reduce cellular oxidative stress in animal models of cholestasis, consistent with the results of the present study [55,56,57]. These results suggest that FRA can significantly induce dysregulation of the amino acid metabolism in zebrafish, resulting in oxidative stress-induced liver damage, suggesting that changes to endogenous metabolites, such as amino acid levels, are potential markers for early monitoring of hepatotoxicity. The results proved that targeted amino acid metabolomics can reveal the changes in amino acid metabolism of zebrafish related to the toxicity caused by FRA. The combination of amino acid metabolomics and the zebrafish model can become a useful tool in hepatotoxicity screening and for finding related drugs.

## 4. Materials and Methods

### 4.1. Materials

FRA was provided by Professor Sujuan Wang (Institute of Materia Medica, Chinese Academy of Medical Sciences, Beijing, China). Ketoconazole (KCZ; HY-B0105) was purchased from MedChemExpress LLC (Monmouth Junction, NJ, USA). Alanine aminotransferase (ALT), aspartate aminotransferase (AST), total bilirubin (TBIL), and direct bilirubin (DBIL) assay kits were purchased from Nanjing Jiancheng Bioengineering Institute (Nanjing, China). The anesthetic tricaine methanesulfonate (MS-222), acridine orange (AO) staining solution, and a Reactive Oxygen Species (ROS) assay kit were purchased from Solarbio Science & Technology Co. Ltd. (Beijing, China). TransScript^®^ Fly First-Strand cDNA Synthesis SuperMix was purchased from TransGen Biotech Co. Ltd. (Beijing, China). KAPA SYBR^®^ FAST qPCR Master Mix (2×) was purchased from Kapa Biosystems (Cape Town, South Africa). Gene-specific primers for qRT-PCR analysis were synthesized by RuiBiotech Biotechnology Co. Ltd. (Beijing, China). Antibodies against JNK (9252T; dilution, 1:1000), p-JNK (4668T; dilution, 1:1000), p53 (2527S; dilution, 1:1000), Bax (5023S; dilution, 1:1000), and cleaved caspase-3 (9661S; dilution, 1:800) were purchased from Cell Signaling Technology (Danvers, MA, USA). A rabbit anti-p-p53 monoclonal antibody (ab223868; dilution, 1:3000) was purchased from Abcam (Cambridge, UK), and a mouse monoclonal antibody against glyceraldehyde 3-phosphate dehydrogenase (GAPDH; ab223868; dilution, 1:2000) was purchased from HUABIO (Woburn, MA, USA). All other reagents were obtained from commercial sources.

### 4.2. Animals

Wild-type zebrafish (strain AB) were purchased from FishBio Co. Ltd. (Shanghai, China) and housed in an aquatic feeding system (Aquaneering, Inc., San Diego, CA, USA) at 28 °C under a 14/10-h day/night photoperiod. Zebrafish embryos were collected after spawning of naturally paired adults and incubated in E3 culture medium (5 mM NaCl, 0.17 mM KCl, 0.33 mM CaCl_2_, and 0.33 mM MgSO_4_) at 28 ± 1 °C [13]. All animal protocols were approved by the Animal Ethics Committee of the Institute of Materia Medica, Chinese Academy of Medical Sciences and Peking Union Medical College (Beijing, China).

### 4.3. Drug Administration

FRA was dissolved in dimethyl sulfoxide (DMSO) to a final concentration of 100 mM. KCZ was dissolved in DMSO to a final concentration of 10 mM. The reserve solution of FRA and KCZ was diluted to the concentration required for each experiment with E3 culture medium (dimethyl sulfoxide content >0.1%). The E3 culture medium was dissolved in distilled water and stored at room temperature.

At 60–72 hpf, the liver of the zebrafish larva begins to grow and develops completely. Up to 120 hpf, the zebrafish larva derives nutrition from the yolk sac, so there is no need to supply food [58,59]. After 48 hpf, healthy zebrafish larvae at the same developmental stage were transferred to the wells of a 6-well plate (10 larvae per well) and supplied with 10 mL of E3 culture medium. At 72 hpf, the E3 culture medium in each well was removed, and 5 mL pre-configured E3 culture medium containing the corresponding concentration of drug solution was added immediately. The zebrafish larvae were continuously exposed to the drug solution for 48 h (120 hpf) to evaluate drug-induced hepatotoxicity.

### 4.4. Determination of Survival Rate

The appropriate drug dosage was determined by calculating the lethal dose for 10% of the population (LD_10_). Each group of 30 zebrafish larvae was transferred to the well of a 6-well plate and exposed to various concentrations of FRA for 120 hpf. During this time, the zebrafish heartbeat was observed using an aquatic biological microscope (SZX-ILLK2002; Olympus Corporation, Tokyo, Japan) and the number of dead zebrafish was counted. The mortality rate of the zebrafish at different concentrations of FRA and the LD_10_ value were calculated using IBM SPSS Statistics for Windows, version 20.0. (IBM Corporation, Armonk, NY, USA).

### 4.5. Microscopic Evaluation of Hepatotoxicity

#### 4.5.1. Liver Gray Value Assay

After 48 h of exposure (120 hpf), the zebrafish larvae were anesthetized by adding 60 μL of 2% MS-222 to each well of the 6-well plate. 10 zebrafish larvae were selected from each group and placed on the slides with grooves. 2% CMC-Na was added to fix the zebrafish and make them maintain the lateral lying position. The liver morphology was observed under an ultra-depth-of-field microscope (VHX-2000; Keyence Corporation, Osaka, Japan). The gray value of the zebrafish liver was measured using Image J software (National Institutes of Health, Bethesda, MD, USA).

#### 4.5.2. AO Staining

After discarding the E3 culture medium, the zebrafish larvae were rinsed 3 times with phosphate-buffered saline (PBS) and then incubated in 5 μg/mL of AO dye for 10 min in the dark [60]. After washing three times with PBS, the zebrafish larvae were anesthetized with 2% MS-222 and placed on glass slides, and the degree of apoptosis of the stained livers was examined using a fluorescence microscope (ZEISS Axio Vert.A1; Carl Zeiss AG, Oberkochen, Germany).

### 4.6. Liver Biochemical Assay

With the sublethal dose (LD10) of FRA as the upper limit, various concentrations were used to evaluate the extent of injury to the zebrafish liver. Each group of 40 zebrafish was homogenized with 0.9% physiological saline solution (1:9 *w*/*v*) in an ice-water bath. The homogenate was centrifuged at 3500 rpm for 10 min, and the supernatant was collected to determine the concentrations of ALT, AST, TBIL, and DBIL using commercially available kits in accordance with the manufacturer’s instructions [54].

### 4.7. Histopathological Analysis

Zebrafish larvae from each group were fixed in 4% paraformaldehyde solution and stored at room temperature. For histopathological analysis, the larvae were dehydrated with graded concentrations of ethanol and embedded in paraffin. The paraffin blocks were cut into 4 μm thick sections, which were stained with H&E, mounted in neutral resin cement, and examined under a microscope (NIKON CI-S; Nikon Corporation, Tokyo, Japan). Pathological changes to the tissue sections were recorded using an imaging system (DS-FI2; Nikon Corporation, Tokyo, Japan).

### 4.8. ROS Assay

Each group of 50 zebrafish larvae was transferred to a centrifuge tube and washed twice with precooled PBS. Zebrafish lumps were cut into granular tissue blocks with ophthalmic scissors and digested with an enzymolysis solution (0.2% collagenase, 0.01% hyaluronidase, 0.002% DNA enzyme, Roswell Park Memorial Institute 1640 medium) (1:9 *w*/*v*) with shaking at 37 °C for 90 min. An equal volume of medium containing serum was added to stop digestion. Then, the tissue precipitant was filtered through a 70-μm screen and the supernatant was collected and centrifuged twice with a medium containing serum at 1200 rpm for 5 min prior to collecting the cell suspension [61]. Then, the fluorescent probe 2′–7′dichlorofluorescin diacetate was added and the cell suspension was incubated at 37 °C for 20 min. Afterward, the ROS content was detected by flow cytometry (FACSVerse; BD Biosciences, San Jose, CA, USA).

### 4.9. qRT-PCR Analysis

Total RNA was extracted from 40 zebrafish larvae in each group using an RNA Rapid Extraction Kit (Shanghai Generay Biotech, Shanghai, China) and reverse-transcribed into complementary DNA using TransScript^®^ Fly RT/RI Enzyme Mix and 2× TS Fly Reaction Mix (TransGen Biotech Co. Ltd.). Amplification by qRT-PCR was conducted using KAPA SYBR^®^ FAST qPCR Master Mix (2×) and gene-specific primers. The mRNA expression levels of all genes were quantified by comparison to the mRNA expression of GAPDH as an internal reference. The gene-specific primers are listed in Table 1.

### 4.10. Western Blot Analysis

Zebrafish larvae from each group (n = 40) were placed in centrifuge tubes, washed 3 times with PBS, homogenized with 300 μL of precooled radioimmunoprecipitation assay (RIPA) super-strong lysis buffer (RIPA lysate:protease inhibitor:phosphatase inhibitor at 50:1:1) for 2 min, and then fractionated with an ultrasonic cell crusher in an ice-water bath. Following centrifugation at 12,000 rpm and 4 °C for 15 min, the protein concentration in the supernatant was determined using the bicinchoninic acid assay. After adjusting the protein concentration with RIPA super-strong lysis buffer, 5× loading buffer was added (1:4 *v*/*v*) to the supernatant, which was then heated at 100 °C for 10 min and stored at −20 °C. The protein samples (30 μg) were separated by electrophoresis using 10% sodium dodecyl sulfate polyacrylamide gels and then transferred to polyvinylidene fluoride membranes, which were blocked with 5% skim milk in Tris-buffered saline with 0.1% Tween^®^ 20 detergent (TBST) at room temperature for 2 h. After washing with TBST, each membrane was incubated overnight with the appropriate diluted primary antibody at 4 °C. The next day, the membranes were washed three times with TBST for 10 min, then incubated with the appropriate diluted secondary antibody at room temperature for 1 h and washed three times with TBST for 10 min. Finally, the specific protein bands were quantified using the MiniChemi™ 610 Mini Chemiluminescent Imaging and Analysis System (Beijing Sage Creation Science Co. Ltd., Beijing, China).

### 4.11. Targeted Amino Acid Metabolomics Test

Zebrafish samples (~10 mg) from each group were placed in centrifuge tubes, mixed with 200 μL of methanol and water (1:1 *v*/*v*), and ground in a grinder at 4 °C for 2 min. After the addition of 1 mL of precooled acetonitrile (with 5% ammonia), the mixture was vortexed for 30 s, ultrasonicated in an ice bath for 30 min, and cooled at −20 °C for 30 min. Then, the samples were centrifuged at 12,000 rpm and 4 °C for 10 min to remove protein precipitates, and 200 μL of the supernatant was dried in a lined tube. Following the addition of 80 μL of methoxylamine hydrochloride pyridine solution (15 mg/mL), the tube was vortexed for 2 min, and the oxime reaction was continued in an oscillating incubator for 90 min at 37 °C. Then, the samples were mixed with 50 μL of N, O-bis(trimethylsilyl)trifluoroacetamide (containing 1% trimethylsilyl chloride) and 20 μL of hexyl hydride, vortexed for 2 min, and reacted at 70 °C for 60 min [28,62]. Afterward, the samples were maintained at room temperature for 30 min prior to metabolomics analysis.

The metabolites’ 19 different amino acids in the samples were quantitatively and qualitatively detected by gas chromatography–mass spectrometry (GC-MS) (TSQ9000; Thermo Fisher Scientific, Waltham, MA, USA) with a DB-5MS capillary column (30 m × 0.25 mm × 0.25 μm, J&W Scientific, Folsom, CA, USA) [63]. The chromatography conditions were as follows: injection volume, 1 μL; carrier gas, high purity helium (purity not less than 99.999%); flow rate, 1.2 mL/min; and temperature of the injection port, 300 °C. The MS conditions were as follows: electron bombardment ion source temperature, 300 °C; transmission line temperature, 280 °C; scanning mode, selective reaction detection scan; and mass scanning range, 40–600 *m*/*z*.

The ion fragments were automatically identified and integrated using TraceFinder 4.1 General Quan software (Thermo Fisher Scientific, Shanghai, China) assisted by manual inspection. Principal component analysis (PCA) score plots and heatmaps were constructed using Metaboanalyst 5.0 software (https://www.metaboanalyst.ca/) (accessed on 24 June 2021) to analyze the multivariate data of amino acid metabolites in zebrafish, which were confirmed by comparing retention times and MS/MS fragmentation patterns with authentic standards of amino acids.

### 4.12. Statistical Analysis

All data are expressed as the mean ± standard deviation (SD) of at least three independent experiments. Data analyses were conducted with one-way analysis of variance and the unpaired Student’s *t*-test (two groups) using IBM SPSS Statistics for Windows, version 20.0. A probability (*p*) value of <0.05 was considered statistically significant.

## 5. Conclusions

The CD extract FRA inhibited expression of bile acid transporters and interfered with mitochondrial membrane potential through the JNK/P53 pathway, resulting in peroxidation of hepatocytes, induction of oxidative stress, and aggravation of cholestatic liver injury. Meanwhile, KCZ can inhibit metabolizing enzyme activities in vivo, thereby alleviating FRA-induced hepatotoxicity in zebrafish. Changes in amino acid levels can be combined with morphological, biochemical, and molecular biology assessments to elucidate the mechanism underlying FRA-induced hepatotoxicity. Monitoring of the amino acid metabolism presents a new approach for the prevention and treatment of DILI.

## Figures and Tables

**Table 1:** qRT-PCR primer sequences.

Gene	Forward Primer (5′-3′)	Reverse Primer (5′-3′)	Gene ID
P-gp	TCGTCATCCTCGCTGTTAGC	ATGACAGCACTTCCTCTGCC	100136865
Bsep	ATTTCCGCAGCAAAGAAGGC	GTTTTTGACCCCGGGAAAGC	571189
Ntcp	TGGTCATCCGCTTGGCTTTA	GAAGGCAGTGAAGGGCATGA	562260
Cyp3a65	AAACCCTGATGAGCATGGAC	CAAGTCTTTGGGGATGAGGA	553969
JNK1	GAGAACTGGTCCTGATGA	TCACCAGATAAACATCCT	65236
p53	GGGCAATCAGCGAGCAAA	ACTGACCTTCCTGAGTCTCCA	30590
Caspase-3	TCAGGCTTGTCGAGGAAC	CTGCCATACTTTGTCATCATTT	140621
GAPDH	ACAGCAACACAGAAGACCGT	GGCAGGTTTCTCAAGACGGA	317743

## Data Availability

Not applicable.

## Sample Availability

Samples of the compounds fraxinellone are available from the Shujuan Wang.
